# Fine motor skills assessment instruments for preschool children with typical development: a scoping review

**DOI:** 10.3389/fpsyg.2025.1620235

**Published:** 2025-09-10

**Authors:** Baifa Zhang, Zhicheng Lin, Chao Li

**Affiliations:** 1 School of Sports, Southwest University, Chongqing, China; 2 School of Sports, Qingdao University, Qingdao, China

**Keywords:** preschooler, children, fine motor skill, instruments, scoping review

## Abstract

**Purpose:**

This study aims to systematically evaluate the available scientific knowledge on fine motor skills assessment instruments for typically developing preschool children.

**Methods:**

Relevant literature was systematically retrieved from Web of Science Core Collection, PubMed, Medline, CNKI, and Wanfang databases from their inception to June 9, 2023. Following the Preferred Reporting Items for Systematic Reviews and Meta-Analyses extension for Scoping Reviews (PRISMA-ScR), included literature was compiled and analyzed.

**Results:**

In total, 58 studies reporting 14 instruments met inclusion criteria: performance-based tests (n = 11), informant-based questionnaires (*n* = 2), and one computer-assisted tool. Based on different types and development purposes, the measurement dimensions and items had varying emphases, and there is no recognized gold standard yet. The reliability of measurements was generally good, while validity needs improvement.

**Conclusion:**

Given the distinct strengths and limitations of available FMS assessment tools, we suggest that selection should align with specific objectives: the ASQ may be most suitable for large-scale screening, the MABC for general FMS assessment in small groups, the Beery VMI for visual-motor integration, and the TIHM for fine motor control assessment.

## 1 Introduction

Early childhood professionals and curricula have long emphasized the significance of fine motor skills (FMS) (Fischer et al., 2020). The Chinese early learning and development guidelines for preschoolers identify FMS as a key dimension in the holistic development of the child. FMS refer to the use of small muscle movements in the hands and fingers to hold or manipulate objects, requiring precise hand-eye coordination (Suggate et al., 2018). These skills can be delineated into visual motor integration (VMI), also referred to as visuomotor or graphomotor skills, and fine motor coordination (FMC), which relate to separate abilities that follow distinct developmental trajectories (Carlson et al., 2013; Flores et al., 2023a). VMI is defined as the ability to integrate visual–spatial stimuli and attention control with fine motor output, requiring greater hand-eye coordination than FMC (Carlson et al., 2013; Flores et al., 2024). This integration emerges from the combined effects of various cognitive and neuromotor processes, including visual–spatial perception, visual size discrimination, visual retrieval, and orientation discrimination (Smits-Engelsman et al., 2001; Dinehart and Manfra, 2013). In VMI assessments, children are typically required to perform tasks involving writing, drawing, cutting, and folding (Grissmer et al., 2010; Martzog et al., 2019). The development of VMI is an important predictor of children’s handwriting skills, with higher VMI levels associated with better performance on literacy tasks (Suggate et al., 2019; Lu et al., 2024). Evidence also supports a link between VMI and mathematical outcomes, with neuroimaging studies revealing overlapping brain networks underlying both visuospatial and numerical processing (Becker et al., 2014). FMC focuses on finger movement flexibility, requiring rapid and accurate finger movements within specified time constraints, such as inserting coins and threading beads—skills also termed non-graphomotor abilities (Carlson et al., 2013; Flores et al., 2023a). FMC assessment tasks fall into three categories, each measured by completion time: (1) translation tasks, which measure the ability to transfer objects between palm and fingertips, such as inserting coins; (2) shift tasks, which assess the ability to manipulate objects through sequential finger movements, such as threading beads; and (3) rotation tasks, which evaluate the ability to rotate objects using finger coordination, such as turning wooden pegs (Pont et al., 2008). Another component of FMC assessment is grasping, which involves adjusting hand movements based on object shape and size to ensure proper manipulation, such as grasping blocks, chopsticks, and writing implements (Strooband et al., 2023). Grasping forms the foundation for developing complex tool-based movements, with assessment measures typically emphasizing process-oriented aspects of movement execution rather than outcome metrics. Researchers have pre-established multi-level scoring criteria based on the developmental trajectories of holding different tools (chopsticks, pens, etc.), analyzing the developmental level of these skills from the perspective of movement developmental sequences, which facilitates specialized evaluation and guidance for preschool children (Burton and Dancisak, 2000; Payne and Isaacs, 2024). Related research has found significant relationships between early FMC and both concurrent and future language development, with FMC demonstrating the ability to predict subsequent language delays (Choi et al., 2018; Bhat et al., 2022).

National education goals identify FMS as a key aspect of school readiness, given that kindergarten children spend between 36 and 66% (with an average of 46%) of their in-class time engaged in fine motor activities, such as writing, cutting, and manipulative play (Marr et al., 2003; Becker et al., 2014). Furthermore, cognitive and fine motor development are closely linked, with established associations between fine motor skills and crystallized intelligence, memory, and fluid reasoning (Davis et al., 2011). Evidence from neuroimaging studies also suggests strong functional coupling between brain regions once thought to support either exclusively cognitive or exclusively fine motor processes (Stoodley, 2012). While FMS play a critical role in early childhood development, research demonstrates that 10–24% of children exhibit developmental delays in this domain, and an additional 13–40% show risk factors for suboptimal skill acquisition (Strooband et al., 2020). Early identification of FMS developmental delays is crucial for timely intervention (Cameron et al., 2012; Oberklaid et al., 2013). Failure to identify and remediate fine motor skill issues in preschoolers in a timely manner may adversely affect their cognitive development and academic achievement in elementary and middle school (Dinehart and Manfra, 2013). Children with fine motor skill impairments commonly experience peer rejection, reduced self-efficacy, and lower self-esteem. These psychological consequences lead to avoidance of activities that highlight their impairments, such as play and social interaction (Gaul and Issartel, 2016). Given the need for timely intervention, effective and reliable assessment tools for FMS are essential (Strooband et al., 2023). Current FMS assessment tools can be broadly categorized into performance-based tests and informant-based questionnaires. Performance-based FMS tests require examiners to evaluate children’s discrete motor abilities through direct observation of structured tasks, using predetermined criteria (Matheis and Estabillo, 2018). These tests require qualified professionals with specialized training in administration, scoring, and interpretation protocols to ensure reliable assessment results, limiting their accessibility primarily to researchers and occupational therapists. Informant-based questionnaires collect FMS ratings from adults who regularly observe the child, such as parents, teachers, and caregivers (Lalor et al., 2016). These tests offer efficiency and cost-effectiveness for large-scale screening, while they are limited by potential observer bias and reduced utility for educational intervention planning (Howard et al., 2019; Chen et al., 2021).

However, the lack of clarity and empirical evidence for distinguishing between the overlapping components of FMS has resulted in considerable variation in how different instruments conceptualize and measure it. Thus, it is difficult for early childhood educators and researchers to know which assessment tool is most appropriate for accurately identifying children who struggle with FMS development. This evaluation is particularly challenging as there is a lack of clarity in the previous review regarding the specific items, validity and reliability of the available FMS measures. A defined literature review is essential to identify effective assessment tools that enable early childhood educators to detect fine motor delays and support children’s development in educational settings. Therefore, this scoping review aimed to systematically map FMS assessment instruments for typically developing preschool children described in the literature. The following research question guided this scoping review: What is the current state of international research on fine motor skills assessment instruments for typically developing preschool children?

## 2 Methods

### 2.1 Search strategy

The main English-language literature databases included Web of Science Core Collection, PubMed, and Medline, while China National Knowledge Infrastructure (CNKI) and Wanfang Database served as the primary Chinese literature sources. The search period covered from database inception to June 9, 2023. The English search terms or keywords consisted of three groups: ① Population: (child* OR preschool OR pre-school* OR boy OR girl) NOT (disorder* OR illness OR disease* OR disab*② Fine motor skills: “fine motor” OR “visual motor” OR graphomot). ③Methodology: tool OR instrument OR assessment OR evaluation OR measurement. Boolean operator “AND” was used between groups in both searches. Additionally, reference lists of included studies were manually reviewed to identify any relevant literature that might have been missed through the database searches.

### 2.2 Identifying relevant studies

This review included full-text articles published in peer-reviewed journals in English or Chinese. Studies were eligible if they involved original research on the development, validation, or application of fine motor skills assessment instruments for preschool children. Participants were limited to typically developing children aged 3–6 years, excluding those with neurodevelopmental conditions (e.g., autism, cerebral palsy). Studies were excluded if they were non-full-text articles, non-English or non-Chinese literature, books, book chapters, unpublished papers, conference proceedings, dissertations, non-empirical research (such as reviews, commentaries, and book reviews), or studies with participants having a mean age below 3 years or above 6 years.

### 2.3 Study selection

Following duplicate removal, two researchers independently screened titles and abstracts for preliminary eligibility. Full texts of potentially eligible studies were then retrieved and reviewed thoroughly. Any disagreements during the screening process were resolved through discussion with a third researcher to determine final inclusion. Information extracted from the included literature comprised: author, country, year, study type, sample characteristics, assessment instruments name, tool type, content measured, evaluation method, reliability and validity, and research findings relevant to the main research questions.

## 3 Results

### 3.1 Characteristics of the included studies

**Figure 1:**
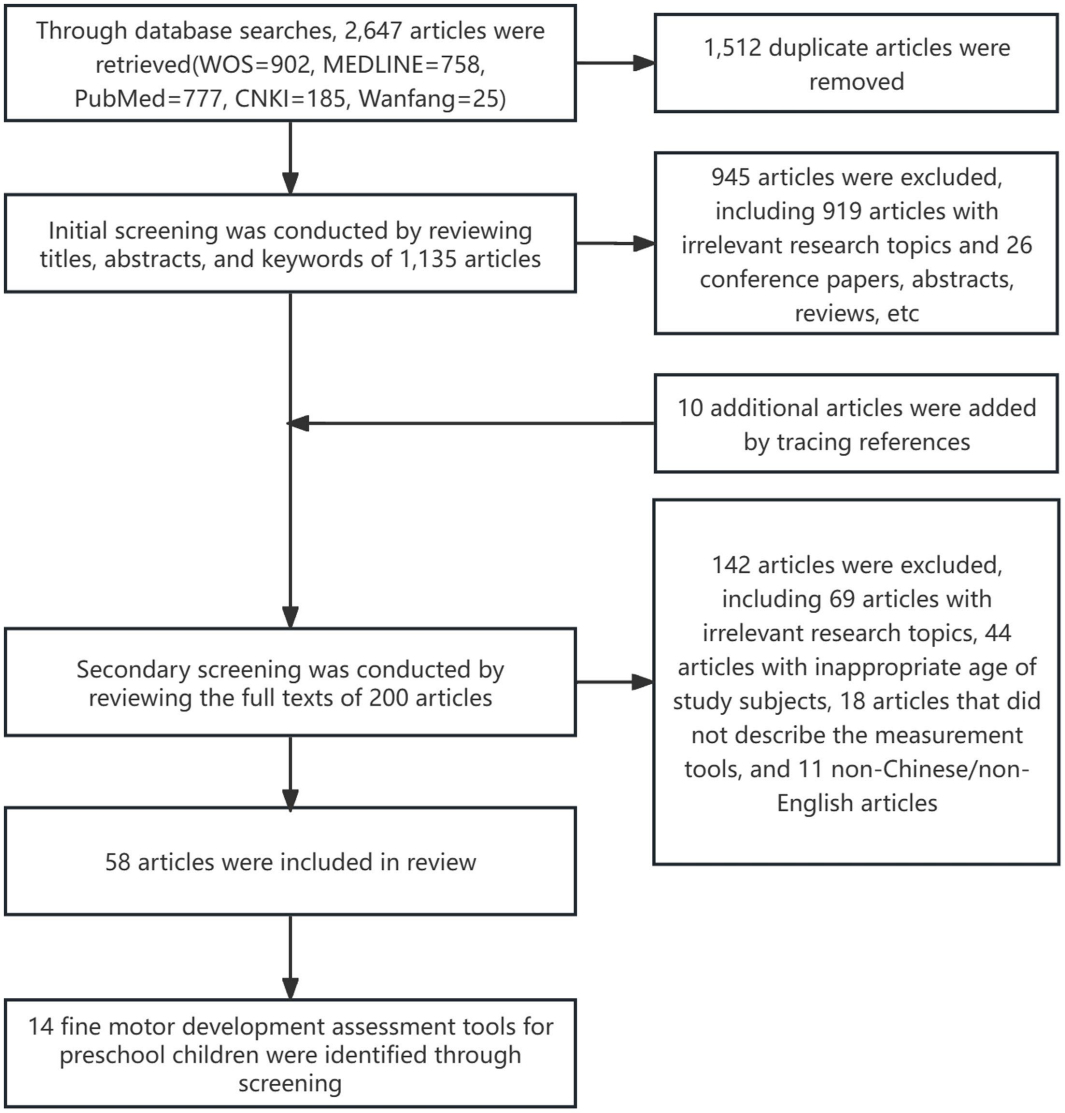
Flowchart of literature screening process.

The initial search yielded 2,647 articles. Following rigorous screening according to the inclusion and exclusion criteria, 58 studies were ultimately included: 20 focused on instruments development and validation, 16 addressed cross-cultural adaptation (localized standardization across different countries), 16 reported cross-sectional investigations, 3 conducted prospective studies, and 3 implemented non-randomized controlled trials. These studies involved 14 different fine motor skills assessment instruments for preschool children, categorized as performance-based tests (*n* = 11), informant-based questionnaires (*n* = 2), and computer-assisted assessments (*n* = 1). These instruments originated from researchers in the United States (*n* = 7), China (*n* = 3), the United Kingdom (*n* = 1), Canada (*n* = 1), Switzerland (*n* = 1), and Australia (*n* = 1), as shown in Table 1. The literature screening process is illustrated in Figure 1.

**Table 1:** Overview of assessment instruments for fine motor skills in preschool children.

Instrument	Abbr.	Format	No. of articles	Country/Region
Ages and stages questionnaires	ASQ	①	12	United States
Developmental motor screening questionnaire	DMSQi	①	1	Taiwan, China
Berry-Buktenica developmental test of visual-motor integration	Beery VMI	②	19	United States
Movement assessment battery for children	MABC	②	6	United Kingdom
Nine hole peg test	9-HPT	②	1	United States
Bruininks-Oseretsky test of motor proficiency	BOT	②	4	Canada
Peabody developmental motor scale	PDMS	②	3	United States
Zurich neuromotor assessment	ZNA	②	2	Switzerland
Functional dexterity test	FDT	②	2	United States
Test of in-hand manipulation	TIHM	②	1	United States
Fine motor growth assessment	FINGA	②	1	Australia
Grip scale	GS	②	1	United States
3–6 years old children’s physical and intellectual evaluation index system	/	②	1	China
Computerized VMI assessment tool using basic strokes	C-VMI	③	1	Taiwan, China

① Informant-based questionnaires. ② Performance-based tests. ③ Computer-assisted assessments.

### 3.2 Characteristics of the FMS assessment instruments

Seven assessment instruments were comprehensive developmental tools that include fine motor skills as one of multiple domains: Ages and Stages Questionnaires (ASQ), Developmental Motor Screening Questionnaire (DMSQi), Movement Assessment Battery for Children (MABC), Bruininks–Oseretsky Test of Motor Proficiency (BOT), Peabody Developmental Motor Scales (PDMS), Zurich Neuromotor Assessment (ZNA), and the Evaluation Index System of Physical and Cognitive Abilities for Children Aged 3–6. Regarding the applicable age range, 8 instruments were specifically designed for preschool children, 4 tools covered school-aged children, and 2 tools extended to adults. In terms of assessment content, the 14 instruments collectively evaluated two categories of fine motor skills: visual motor integration (*n* = 9) and fine motor coordination (*n* = 12). Regarding administration time, 11 tools reported testing duration. The PDMS required the most time (20–30 min), while the Nine-Hole Peg Test (9-HPT) required the least (1–3 min). Across cross-sectional, prospective, and non-randomized controlled studies, the most frequently used assessment tools were the Beery–Buktenica Developmental Test of Visual–Motor Integration (Beery VMI, *n* = 10), MABC (*n* = 5), and ASQ (*n* = 4) (Table 2).

**Table 2:** Basic characteristics of assessment instruments for fine motor skills in preschool children.

Instrument	Age range	Categories	Measurement tasks	Duration	Literature source
ASQ	0:1 ~ 5:6 y	①	Cutting paper, copying figures, drawing within specific boundaries, etc.	<10 min	A (Heo et al., 2008; Kerstjens et al., 2009; Charafeddine et al., 2013; Filgueiras et al., 2013; Wei et al., 2015; Hsiao et al., 2017; van Heerden et al., 2017; Ramos and Barba, 2021) B (Rubio-Codina et al., 2016) C (Wei et al., 2011; Yang et al., 2018) E (Olhaberry et al., 2019; Williams and Johnson, 2022)
		②	Grasping blocks, pencils, etc.		
DMSQi	0:3 ~ 6:0 y	①	Copying figures, folding paper, etc.	<10 min	B (Chen et al., 2021)
		②	Grasping buttons, blocks, chopsticks, etc.		
Beery VMI	2:0 ~ 18:0 y	①	Copying figures, drawing within specific boundaries	<10 min	A (Shi et al., 1995; Mao et al., 1999; Lan and Li, 2001; Josman et al., 2006; Lim et al., 2015; Ng et al., 2015) B (Avi-Itzhak and Obler, 2008; Brown and Rodger, 2008; Brown et al., 2009) C (Lu and Zhao, 1996; Hu et al., 1999; Zhang and Lin, 2001; Li et al., 2002; Daly et al., 2003; Xu et al., 2019; Coetzee et al., 2020) D (Marr and Cermak, 2002; Bart et al., 2007) E (Sommerfeld et al., 2021)
MABC	3:0 ~ 16:0 y	①	Drawing within specific boundaries	<5 min	A (Hua et al., 2012) B (Van Der Veer et al., 2021) C (Kokštejn et al., 2017; Ma et al., 2019; Maurer and Roebers, 2020; Zhang et al., 2020; Zheng and Sun, 2022)
		②	Inserting coins, stringing beads		
9-HPT	2:0 ~ 99:0 y	②	Pegboard	< 5 min	B (de Vries et al., 2015)
BOT	4:0 ~ 21:11 y	①	Copying figures, throwing/catching tennis balls, hitting tennis balls	/	A (Kambas and Aggeloussis, 2006; Chui et al., 2007) B (Venetsanou et al., 2009; Gharaei et al., 2019)
		②	Picking up coins, pegboard, stringing beads, sorting cards, dotting circles		
PDMS	0:0 ~ 5:0 y	①	Copying figures, drawing within specific range, cutting paper, folding paper, etc.	20–30 min	A (Rebelo et al., 2021) B (Chien and Bond, 2009) D (Darrah et al., 2009)
		②	Finger-to-finger touching, grasping towels, pellets, blocks, buttons, pencils, etc.		
ZNA	3:0 ~ 18:0 y	②	Turning screws, pegboard, stringing beads	<20 min	C (Kakebeeke et al., 2017, 2018)
FDT	3:0 ~ 69:0 y	②	Pegboard	<2 min	B (Tissue et al., 2017; Tremblay et al., 2019)
TIHM	3:0 ~ 6:0 y	②	Pegboard	5–7 min	B (Pont et al., 2008)
FINGA	3:0 ~ 5:0 y	①	Copying figures, drawing within specific boundaries, folding paper, cutting paper	14–20 min	B (Strooband et al., 2023)
		②	Stringing beads, stacking blocks, assembling Lego		
		②	Grasping pencils		
GS	3:0 ~ 6:0 y	②	Grasping pencils	/	B (Burton and Dancisak, 2000)
3–6 Years Old Children’s Physical and Intellectual Evaluation Index System	3:0 ~ 6:0 y	① ②	Drawing within specific boundaries, copying figures, dotting within circles, inserting coins, stringing beads	/	B (Lv et al., 2022)
C-VMI	4:0 ~ 6:0 y	①	Copying Chinese character strokes	/	B (Li et al., 2018)

①, Visual-motor integration; ②, Fine motor coordination; A, Local standardization; B, Tool development and validation; C, Cross-sectional survey; D, Prospective survey; E, Non-randomized controlled trial; ASQ, Ages And Stages Questionnaires; DMSQi, Developmental Motor Screening Questionnaire; Beery VMI, Berry-Buktenica Developmental Test of Visual-Motor Integration; MABC, Movement Assessment Battery for Children; 9-HPT, Nine Hole Peg Test; BOT, Bruininks-Oseretsky Test of Motor Proficiency; PDMS, Peabody Developmental Motor Scale; ZNA, Zurich Neuromotor Assessment; FDT, Functional Dexterity Test; TIHM, Test of In-Hand Manipulation; FINGA, Fine Motor Growth Assessment; GS, Grip Scale; C-VMI, Computerized VMI Assessment Tool Using Basic Strokes.

### 3.3 Reliability and validity of the FMS assessment instruments

Regarding reliability testing, 8 tools were validated for internal consistency, using Cronbach’s *α* and split-half reliability as measurement indicators. Eight instruments were validated for test–retest reliability, using the Intraclass Correlation Coefficient and Pearson correlation coefficient as measurement indicators. Five instruments were validated for inter-rater reliability, using Kappa coefficient, Intraclass Correlation Coefficient, and Pearson correlation coefficient as indicators. For validity testing, 8 instruments were validated for criterion validity. Measurement indicators included true positive rate, Pearson correlation coefficient, and differences in area under the ROC curve between the target tool and external criteria. External criteria included the Bayley Scales of Infant and Toddler Development, Denver Developmental Screening Test, Beijing Gesell Developmental Schedule, Test of Visual Perceptual Skills, Wechsler Intelligence Scale for Children, Developmental Coordination Disorder Questionnaire, assessment tools for writing readiness, and the Taylor Hand Function Test. Six instruments were validated for construct validity, with measurement indicators including Infit Mean Square from Rasch analysis, chi-square to degrees of freedom ratio (χ^2^/df), and Comparative Fit Index (CFI) from confirmatory factor analysis. Additionally, 5 instruments were introduced and standardized across multiple countries, with reliability and validity testing conducted in typically developing preschool populations. These tools include ASQ, Beery VMI, MABC, PDMS, and BOT. Among them, the first four were introduced by Chinese researchers and applied to fine motor development assessment of typically developing preschool children in China (Table 3).

**Table 3:** Reliability and validity of assessment instruments for fine motor skills in preschool children.

Tool name	Revision country (Region)	Internal consistency	Test–retest reliability	Inter-rater reliability	Criterion validity	Construct validity
ASQ	United States (Rubio-Codina et al., 2016)	α = 0.57	ICC = 0.37	/	*r* = 0.08–0.38 Bayley	/
	Brazil (Filgueiras et al., 2013)	α = 0.64–0.89	/	/	/	$\chi^{2}/\mathrm{df}$ = 3.50
	Korea (Heo et al., 2008)	α = 0.42–0.90	/	/	tp = 0.75–1.00 Denver	/
	South Africa (Hsiao et al., 2017)	α = 0.20–0.79	/	/	/	/
	China (Wei et al., 2015)	α = 0.80	*r* = 0.80	*r* = 0.80	tp = 0.85 Beijing Gesell	/
DMSQi	Taiwan, China (Chen et al., 2021)	α = 0.97–0.98	ICC = 0.95	/	*r* = 0.85–0.91 PDMS	/
Beery VMI	United States (Brown and Rodger, 2008; Brown et al., 2009)	s = 0.82–0.92	ICC = 0.92	*r* = 0.92–0.98	*r* = 0.62–0.75 DTVP	MnSq = 0.77–1.30
	China (Shi et al., 1995; Lan and Li, 2001)	s = 0.93–0.96	*r* = 0.96–0.97 ICC = 0.90	*r* = 0.95 *k* = 0.81	*r* = 0.60–0.69 WISC-R	/
	Hong Kong, China (Ng et al., 2015)	/	/	/	/	MnSq = 0.68–1.46
	Taiwan, China (Mao et al., 1999)	/	/	/	/	MnSq = 0.63–1.18
MABC	United States (Van Der Veer et al., 2021)	/	/	/	*r* = 0.47 ZNA	/
	China (Hua et al., 2012)	α = 0.50	ICC = 0.94	/	ROC = 0.18 DCDQ	$\chi^{2}/\mathrm{df}$ = 5.94
9-HPT	United States (de Vries et al., 2015)	/	/	/	*r* = 0.40 WRITIC-TP	/
BOT	United States (Gharaei et al., 2019)	/	ICC = 0.80	/	*r* = 0.89 MABC	/
	Greece (Kambas and Aggeloussis, 2006)	α = 0.87	/	/	/	/
PDMS	Taiwan, China (Chien and Bond, 2009)	/	/	/	/	MnSq = 0.60–2.79
	Portugal (Rebelo et al., 2021)	α = 0.69	ICC = 0.71	/	/	CFI = 1.00
ZNA	United States	/	/	/	/	/
FDT	United States (Tissue et al., 2017; Tremblay et al., 2019)	/	ICC = 0.90	ICC = 0.89–0.99	*r* = 0.66–0.67 JHFT	/
TIHM	United States (Pont et al., 2008)	/	/	/	/	MnSq = 0.72–1.47
FINGA	United States (Strooband et al., 2023)	α = 0.94	/	/	*r* = 0.84 PDMS	/
GS	United States (Burton and Dancisak, 2000)	/	/	*r* = 0.67	/	/
3–6 Years Old Children’s Physical and Intellectual Evaluation Index System	China (Lv et al., 2022)	α = 0.81	*r* = 0.95	*r* = 0.96	/	$\chi^{2}/\mathrm{df}$ =1.39
C-VMI	Taiwan, China (Li et al., 2018)	/	/	/	/	/

α, Cronbach’s alpha coefficient; s, split-half reliability; ICC, Intraclass Correlation Coefficient; k, Kappa coefficient; r, Pearson correlation coefficient; χ^2^, chi-square/degrees of freedom ratio; tp, true positive percentage (accuracy); MnSq, Infit Mean Square (weighted mean squared residual); ROC, difference in area under the ROC curve between the tool and external criterion; CFI, Comparative Fit Index; Bayley, Bayley Scales of Infant and Toddler Development; Denver, Denver Developmental Screening Test; DTVP, Developmental Test of Visual Perception; WISC-R, Wechsler Intelligence Scale for Children-Revised; DCDQ, Developmental Coordination Disorder Questionnaire; WRITIC-TP, Writing Readiness Inventory Tool In Children-Task Performance; JHFT, Jebsen-Taylor Hand Function Test; ASQ, Ages And Stages Questionnaires; DMSQi, Developmental Motor Screening Questionnaire; Beery VMI, Berry-Buktenica Developmental Test of Visual-Motor Integration; MABC, Movement Assessment Battery for Children; 9-HPT, Nine Hole Peg Test; BOT, Bruininks-Oseretsky Test of Motor Proficiency; PDMS, Peabody Developmental Motor Scale; ZNA, Zurich Neuromotor Assessment; FDT, Functional Dexterity Test; TIHM, Test of In-Hand Manipulation; FINGA, Fine Motor Growth Assessment; GS, Grip Scale; C-VMI, Computerized VMI Assessment Tool Using Basic Strokes.

## 4 Discussion

### 4.1 Informant-based questionnaires

There are two questionnaire instruments used for assessing preschool children’s fine motor skills (Table 1), among which the Ages and Stages Questionnaire (ASQ) is more widely applied. ASQ was developed in 1995 by the Human Development Center and Early Intervention Research Institute at the University of Oregon, with the latest version ASQ-3 released in 2009. It is suitable for children aged 1–66 months and focuses on screening for early developmental delays, with the child’s caregiver serving as the proxy reporter. ASQ is divided into 21 age groups, with each age group questionnaire containing 30 questions evaluating children’s developmental progress from five comprehensive aspects: communication, gross motor, fine motor, problem-solving, and personal-social skills. The fine motor assessment comprises six questions related to VMI and FMC, with response options of “yes” (10 points), “sometimes” (5 points), and “no” (0 points). The sum of the six scores constitutes the fine motor development score. ASQ has been translated into multiple languages and widely used worldwide (Heo et al., 2008; Filgueiras et al., 2013; Hsiao et al., 2017), demonstrating good reliability and validity. It was introduced to China by Bian Xiaoyan, who completed its localization standardization and normative reliability and validity testing (Wei et al., 2015). This questionnaire has numerous advantages, including simplicity, speed, low cost, and flexibility, making it suitable for large-scale child screening. However, it provides too little information and cannot eliminate the subjective tendencies of caregivers when evaluating children.

Furthermore, Chen et al. (2021) designed the Developmental Motor Screening Questionnaire (DMSQi) as a culturally adapted, parent- or caregiver-reported screening tool for motor development in children aged 3 months to 6 years. The purpose of the DMSQi is to enable clinicians to identify children with motor development delays early. The DMSQi consists of 78 items, with 42 measuring the fine motor domain across VMI and FMC subdomains. Items are scored on a four-point scale (1–4) reflecting developmental progression: not yet developed (1), beginning (2), intermediate (3), and proficient (4). The complete assessment requires approximately 10 min for administration. The fine motor domain and its subdomains of the DMSQi demonstrated excellent internal consistency, with Cronbach’s alpha values ranging from 0.94 to 0.97, and strong test–retest reliability (ICC = 0.95). Concurrent validity assessment using the PDMS as the reference standard demonstrated strong correlation coefficients ranging from *r* = 0.85 to 0.91 (Chen et al., 2021). A notable strength of the DMSQi is its integration of illustrated and textual descriptions for each item, facilitating more accurate and efficient item interpretation compared to text-only formats. The recruitment of participants exclusively from New Taipei City constitutes a limitation, potentially restricting the generalizability of findings to broader populations of typically developing children.

The structure of preschool children’s fine motor skills is relatively complex, and the measurement dimensions varying according to assessment instruments and developmental objectives. The informant-based questionnaires included in this review assess grasping components of FMC but lack evaluation of other essential FMC components. This is because informant-based questionnaires are widely used for large-scale, rapid screening of children with developmental delays to facilitate early intervention. Since proxy reporters are typically children’s caregivers, these instruments’ items are more closely aligned with daily life situations (e.g., “Can your child unbutton multiple buttons?”), thereby reducing measurement errors due to information asymmetry from caregivers. In contrast, other fine motor control components (e.g., turning pegs, threading beads) require specific operational contexts and standardized tools, thereby presenting significant challenges for data collection.

### 4.2 Performance-based tests

#### 4.2.1 Fine motor assessments

There are 11 performance-based tests used for assessing typical developing preschool children’s fine motor skills, which fall into two categories. The first category comprises specialized fine motor assessments: the Beery-Buktenica Developmental Test of Visual-Motor Integration (Beery VMI), Nine-Hole Peg Test (9-HPT), Test of In-Hand Manipulation (TIHM), Functional Dexterity Test (FDT), Grip Scale (GS), and Fine Motor Growth Assessment (FINGA). Among these instruments, the Beery VMI is the most widely used. Originally developed by Beery in 1967, it has been revised repeatedly, with the sixth edition (Beery VMI-6) released in 2010. The Beery VMI is standardized for individuals aged 2 years through adulthood and primarily assesses VMI abilities during critical developmental stages. The Beery VMI employs a geometric figure-copying format comprising 30 dichotomously scored items, with a 21-item short form available for children aged 2 to 7 years that requires approximately 10 min for administration (Flores et al., 2023b). Additionally, Beery VMI includes two supplementary tests for visual perception and motor coordination, each taking 5 min to administer. Marr and Cermak (2002) and Brown et al. (2009) conducted comprehensive reliability and validity testing for Beery VMI, using split-half reliability, ICC, correlation coefficients, and MnSq values to determine Beery VMI’s internal consistency, test–retest reliability, inter-rater reliability, and construct validity. Research shows that Beery VMI’s split-half reliability ranges between 0.82–0.92, test–retest reliability ICC value is 0.92, correlation coefficients range between 0.92–0.98, and MnSq values range between 0.77–1.30, indicating that Beery VMI has good psychometric properties. Due to its unique testing format, which is not limited by cultural or educational level, Beery VMI has good cross-cultural adaptability and has been widely used in different countries and linguistic-cultural environments (Josman et al., 2006; Lim et al., 2015). Chinese scholars have introduced this tool in Taiwan (Mao et al., 1999), Hong Kong (Ng et al., 2015), and mainland China (Lu and Zhao, 1996; Hu et al., 1999; Lan and Li, 2001; Zhang and Lin, 2001; Xu et al., 2019) and completed standardization research for urban children in Shaanxi Province (Shi et al., 1995). However, there is no Chinese normative study for Beery VMI-6 yet. Beery VMI’s advantages lie in its testing convenience and cross-cultural applicability, but the tool mainly evaluates visual motor integration skills and cannot comprehensively reflect children’s fine motor development level.

The 9-HPT, TIHM, and FDT are timed pegboard-based assessments of FMC, with only the TIHM specifically designed for children aged 3 to 6 years (Aaron and Jansen, 2003; Pont et al., 2008; Feys et al., 2017). The three pegboard tests demonstrate efficient administration times: the 9-HPT requires less than 5 min, the TIHM takes 5 to 7 min for administration and scoring, and the FDT requires 15 s to 2 min (Smith et al., 2000; Pont et al., 2008). The 9-HPT and TIHM use a 9-hole pegboard, whereas the FDT uses a board with 16 peg holes arranged in a 4 × 4 grid. The 9-HPT requires children to insert nine pegs individually into a pegboard and subsequently remove them, completing two trials with the dominant hand. The faster trial time serves as the assessment score. The TIHM test comprises five tasks. The first task requires children to use their fingertips to rotate five figurines 180 degrees onto their heads and return them to their original holes. Tasks 2 through 5 are translation-with-stabilization activities in which children pick up two, three, four, or five pegs, respectively, using their fingertips, transfer them to the palm, and replace them in the pegboard. Performance measures include completion time, number of dropped or externally stabilized pegs, and quality of in-hand manipulation skills. The FDT measures completion time in seconds for one-handed peg turning. Five-second penalties are added for each instance of compensatory supination or board contact for assistance. When a peg is dropped, a 10-s penalty is applied and timing is paused. The child retrieves the peg, replaces it in its unturned position, and continues from that point, with timing resumed from where it stopped. These assessment tools are efficiently administered, procedurally straightforward, and commercially available. The TIHM’s assessment of both translation and rotation components of FMC, unlike the 9-HPT and FDT which measure only rotation, better correlates test results with children’s everyday functional abilities (Pont et al., 2009).

The Grasp Scale (GS), developed by Schneck, evaluates grasping aspects of FMC through 10 developmentally ordered patterns. The GS is recommended for documenting individual children’s grip patterns and their developmental changes, as well as for designing and evaluating interventions (Burton and Dancisak, 2000). However, it cannot evaluate other essential grasping skills required by the Chinese Learning and Development Guidelines for Children Aged 3–6, such as the proper use of scissors and chopsticks.

Strooband et al. (2023) developed the Fine Motor Growth Assessment (FINGA), an observational tool that measures both FMC and VMI in children aged 3 to 5 years by rating their performance on two standardized tasks: an individual paper plane building task and a group card copying task. This test takes approximately 14–20 min to complete. The test offers two primary advantages: its clear alignment with activities naturally occurring within early childhood education contexts and its direct application in informing evidence-based planning for children’s learning and development. The limitation of FINGA is that it has not yet been validated when administered by early childhood educators in practice.

#### 4.2.2 Subscales of fundamental motor skill assessment

The second category of performance-based tests includes subscales of fundamental motor skill assessments, such as the Zurich Neuromotor Assessment (ZNA), Bruininks-Oseretsky Test (BOT), Peabody Developmental Motor Scales (PDMS), Movement Assessment Battery for Children (MABC), and the Evaluation Index System of Physical and Cognitive Abilities for Children aged 3 to 6. The fine motor subscales of the ZNA, MABC, BOT, and PDMS are internationally recognized assessment tools for evaluating fine motor skills, requiring testers to possess foundational knowledge of test theory and principles, standardized training, and familiarity with the child participant’s background (Cools et al., 2009). The ZNA measures FMC exclusively, while the MABC, BOT, and PDMS assess both FMC and VMI. The PDMS is specifically designed for children aged 0 to 6 years, while the remaining instruments encompass broader age ranges spanning both preschool children and adolescents. For children in the 3–6 year age band, the MABC provides differentiated content and the ZNA reduces the number of repetitions, whereas the BOT employs identical tasks across all ages. The MABC is the most frequently used instrument among the aforementioned measures, comprising the fewest assessment items and requiring the shortest administration time, typically under 5 min (Flores et al., 2023b).

Lv et al. (2022) recently developed an assessment instrument, the Evaluation Index System of Physical and Cognitive Abilities for Children aged 3–6. The fine motor subscale of this assessment instrument comprises four tasks: Dotting in Circles, Tracing a Path, Bead Stringing, and Figure Copying. It demonstrated satisfactory internal consistency (Cronbach’s *α* = 0.81), strong test–retest reliability (*r* = 0.95), and strong inter-rater reliability (*r* = 0.96). However, the validation samples for this instrument were limited to 4- to 6-year-old children from the Shanghai region, excluding 3-year-old children and lacking national representation, thereby limiting generalizability.

### 4.3 Computer-assisted assessment tools

The Computerized VMI Assessment Tool Using Basic Strokes (C-VMI), developed by the National Taichung University of Education in Taiwan, is used to measure visual motor integration ability in Taiwanese preschool children, focusing on screening and intervention for children with insufficient writing readiness (Li et al., 2018). C-VMI includes 34 basic strokes announced by the Taiwan education department, requiring children to control an electronic pen on a touch panel to copy the strokes. It completes scoring by real-time analysis of stroke parameters (such as path length, average speed, pressure, presence of pauses or tremors, etc.) to determine whether children have writing difficulties. The developers recruited 551 preschool children in Taiwan for validity testing, using the evaluation results from preschool education experts as validity criteria, and found that the tool has good validity (true positive rate = 68.1% ~ 90.2%). C-VMI’s advantages lie in its objective and standardized assessment process, simplicity, and convenience, with test content conforming to Chinese local culture. However, this tool has only been applied in research with Taiwanese preschool children and has not been used in studies in other regions. Additionally, it mainly evaluates children’s visual motor integration ability and cannot comprehensively assess children’s fine motor development level.

### 4.4 Reliability and validity

An important prerequisite for motor development research is ensuring the reliability and accuracy of assessment tools. Reliability refers to the dependability of tests, manifested as stability and consistency of test results. Most instruments included in this review demonstrated good internal consistency (*α* > 0.65 or s > 0.7) and test–retest reliability (ICC > 0.60) but lacked data on inter-rater reliability. Among them, ASQ, Beery VMI, and the Evaluation Index System of Physical and Cognitive Abilities for Children Aged 3–6 underwent the most comprehensive reliability testing in Chinese preschool populations, covering internal consistency, test–retest reliability, and inter-rater reliability. Notably, MABC, frequently used by Chinese researchers (Hua et al., 2012; Ma et al., 2019; Zhang et al., 2020) for assessing preschool children’s fine motor skills, includes only three relevant test tasks (threading beads, inserting coins, and drawing tasks) that measure two distinct fine motor domains (FMC and VMI). This results in questionable internal consistency, requiring appropriate revisions. For instance, Hua et al. (2012) found that removing the drawing task significantly improved the task homogeneity of MABC. Inter-rater reliability, as an important indicator for ensuring standardized test administration, is crucial for verifying the stability of process-oriented assessment tools. Grasping tasks for assessing FMC represent typical process-oriented evaluation methodologies. However, among the instruments that incorporate such tasks examined in this study, only the ASQ and GS reported inter-rater reliability, with the GS demonstrating values far below the acceptable threshold (*r* > 0.85). Burton and Dancisak (2000) attributed GS’s low inter-rater reliability to complex grading standards for different pencil grips and lack of example guidance, increasing identification difficulty for testers.

Therefore, the measurement performance of grasping tasks assessment tools in preschool populations still requires further validation. Validity refers to the degree of consistency between actual and expected results of a test or scale, reflecting the accuracy and scientific nature of measured results. Regarding validity testing of the tools included in this review, except for Beery VMI and MABC, most assessment tools have not undergone comprehensive validity testing in typically developing preschool populations, relying solely on either construct validity or criterion validity testing methods. Construct validity, an important indicator for verifying the degree of conformity between assessment tool measurement dimensions and the construct dimensions being measured, is now tested using Rasch models from Item Response Theory rather than being limited to statistical methods from Classical Test Theory, particularly for cross-cultural adaptation. Chien and Bond (2009) tested the construct validity of PDMS in Taiwanese children using the Rasch model and found that four grasping tasks items showed poor fit and ceiling effects, indicating the necessity of revising this tool for use with Chinese children. Additionally, due to the lack of a recognized effective gold standard, the external criteria for tools included in this review are diverse, with significant differences in assessment content and indicators, hindering horizontal comparison of criterion validity between different tools and their widespread promotion. For example, cross-cultural adaptation studies of ASQ (Heo et al., 2008; Wei et al., 2015; Rubio-Codina et al., 2016) reported criterion validity using Denver, Beijing Gesell, and Bayley tools as external criteria, showing polarized correlations between ASQ and these three types of criteria.

## 5 Conclusion

This scoping review conducted a comprehensive search and analysis of literature related to fine motor skills assessment instruments for preschool children. The findings reveal that Performance-based tests predominate among preschool fine motor skills assessment instruments, with varying emphases on measurement dimensions and items. There is currently no recognized gold standard, and the overall validity of existing tools requires improvement. Given that FMS assessment tools each offer distinct strengths and limitations, tool selection should align with specific research objectives. For large-scale screening of early developmental delays, the ASQ is widely recommended given its simplicity, speed, low cost, and flexibility, as evidenced by its translation into multiple languages and extensive worldwide implementation. For assessing general FMS in small preschool groups, the internationally recognized MABC is particularly recommended, as it measures both FMC and VMI using the fewest items and shortest administration time among comparable instruments. When the focus is specifically on VMI, the widely used Beery VMI is recommended given its combination of good psychometric properties, convenience, and demonstrated cross-cultural applicability. For FMC specific assessments, the commercially available TIHM is recommended because it evaluates multiple FMC components including translation and rotation, is designed for children aged 3–6 years, and offers efficient administration with straightforward procedures.

## Data Availability

The original contributions presented in the study are included in the article/supplementary material, further inquiries can be directed to the corresponding author.
